# Psychosocial burden and healthcare disillusionment in recurrent UTI: a large-scale international survey of patient perspectives

**DOI:** 10.3389/fruro.2023.1264299

**Published:** 2023-09-20

**Authors:** Kayleigh Maxwell, Lindsey Roberts, Melissa Kramer, Jessica Price, Abigail Newlands, Katherine A. Finlay

**Affiliations:** ^1^ Faculty of Natural Sciences, University of Stirling, Stirling, United Kingdom; ^2^ School of Psychology, University of Buckingham, Buckingham, United Kingdom; ^3^ Live UTI Free Limited, Dublin, Ireland; ^4^ School of Psychology and Clinical Language Sciences, University of Reading, Reading, United Kingdom

**Keywords:** urinary tract infections, chronic illness, burden of illness, quality of healthcare, patient-centered care

## Abstract

**Objectives:**

Recurrent UTI (rUTI) is a debilitating health condition that is associated with persistent mental, physical, and social burdens. People living with rUTI face inconsistencies in diagnostic testing and fragmented treatment pathways alongside their symptoms, which are likely to add considerably to their illness-related burdens. This study aimed to characterize the factors negatively impacting this population using the qualitative perspectives of people living with the condition.

**Methods:**

Qualitative data were collected via free-text responses using an online survey hosted by an rUTI patient advocacy website. Female participants with self-reported rUTI (*n* = 1,983) described the factors that were most salient to their experience of living with the condition. Data were analyzed using a coding reliability approach to thematic analysis.

**Results:**

Two overarching themes were identified: (1) the patient burden of rUTI, which describes the multifaceted biopsychosocial impact of the illness, and (2) healthcare disillusionment, which describes patient dissatisfaction with healthcare received, both in terms of the treatments offered and communication with healthcare professionals. The patient burden of rUTI encompassed four subordinate themes: facing ongoing uncertainty; symptom salience; sex is not simple anymore; and perceived UTI stigma. Healthcare disillusionment included three subordinate themes: discomfort with frequent antibiotic use; fragmented treatment pathways; and devalued patient perspectives.

**Conclusions:**

The findings demonstrated that ambiguity in the diagnosis of rUTI and inconsistencies in the subsequent treatment pathway are exacerbated by poor patient–clinician communication. The extent of the female-specific burden of rUTI symptoms confirmed the harmful effects of illness-related stigma. This novel qualitative reporting of rUTI symptom burden and life impact highlights the urgent need for increased patient-centered care for those living with rUTI. More effective rUTI management could have a major impact on treatment outcomes and patient-reported psychosocial wellbeing.

## 1 Introduction

Urinary tract infection (UTI) (infection of the bladder, ureters, kidneys, and/or urethra) is one of the most common bacterial infections in humans (1). Predominantly a women’s health issue, UTI has an annual incidence rate of 10% in adult women, compared with 0.1% in adult men (2). UTIs affect more than 400 million people worldwide annually, with an associated estimated healthcare expenditure of $1.6 billion every year in the United States alone (3, 4). Common symptoms include a frequent and overwhelming urge to urinate and painful urination (5). Approximately 30%–50% of women who are affected will experience recurrence within 12 months (6). Recurrent UTI (rUTI) is defined at least two infections experienced within the previous 6 months, or at least three within the previous 12 months (7, 8). Due to the recurrent nature of this condition, in addition to great symptom burden, which is defined as the severity and impact of symptoms, living with rUTI can have a detrimental effect on quality of life (9, 10). However, the patient-reported experience of rUTI is underresearched. Quantitative research has shed light on the effect of rUTI on patients’ mental health and physical functioning (11, 12). However, qualitative research in this area has either focused on acute UTI or rUTI in specific populations, such as in people with spinal cord injury or pregnant women (13), and has prioritized the exploration of attitudes toward antimicrobial use (14, 15). There is an urgent need, therefore, to highlight the patient-reported qualitative experience of living with rUTI, taking a more representative perspective.

It is clear that living with rUTI poses a sizable challenge to the mental health, physical functioning, social relationships, and financial stability of patients. Quantitative mental health research has found that rUTI patients score highly on scales of depression, anxiety, and sexual distress (11, 12, 16), with approximately 70% of patients experiencing depressive symptoms (11). It has been found that rUTI impairs sexual functioning in over 78% of patients (17), with 80% reporting both poor sexual function and high levels of sexual distress (12). People living with rUTI typically score poorly on both sexual functioning and general social functioning scales (10, 16, 18). This reflects the likelihood that rUTI inhibits the maintenance of social relationships and daily activities. A 6-week study found that those with UTI recurrence were three times more likely to be unable to complete normal daily activities than those with resolved UTI and reported an average of 22 hours spent in bed (i.e., absent from work) due to UTI-related illness (19). This negative impact on working life, in combination with the substantial cost of various UTI treatments (20), creates a financial burden for rUTI patients. It is therefore clear that the burden of rUTI encompasses not only symptom severity but also severe psychosocial implications, with women being disproportionately affected.

These burdens may be exacerbated by current treatment pathways for UTI recurrence. Well-established testing methods such as urine culture have been criticized for focusing on a narrow range of bacteria (21) and more advanced testing methods accounting for other uropathogenic bacteria are difficult to elicit from healthcare professionals without a special interest in urology. In addition, UTIs share a similar symptomatology to other urogenital tract infections, making it challenging to accurately diagnose them (22). Repeat antibiotic treatment can also be problematic as it can exacerbate antimicrobial resistance (23). With research on non-antibiotic management strategies now increasingly in the public consciousness (24), patients want to be able to have conversations with their doctors about long-term options (14, 15). However, many patients report barriers to having their experiences acknowledged and validated by medical professionals (18), which can result in a lack of patient-centered care and limit shared decision-making about treatment strategies. Gender-based bias in doctor–patient interactions may exacerbate these barriers further, with female patients most dissatisfied with their care, an issue that is mitigated only partially if their medical consultation is with a person-centered female doctor (25). However, as female urologists constitute a small proportion of the workforce (only 10% in the United States (26)), the probability of female patients with rUTI being supported by female clinicians is low, further diminishing the likelihood of their feeling satisfied with their treatment. It is evident that there is a need to gain a clearer understanding of how care delivery for rUTI can be enhanced.

The current study therefore aimed to explore how rUTI is experienced by gathering the viewpoints of people with self-reported rUTI. To add weight to the understanding of individual experiences of the condition, large-scale qualitative survey data were used to explore commonalities. Given how little is known about the patient experience related to this condition, this research aimed to give patients the opportunity to explain factors around living with rUTI that were significant to them. Understanding the importance of the patient experience in this population would enable traditional testing and treatment methods to be enhanced through recognition of rUTI burden.

## 2 Materials and methods

### 2.1 Study design and participant recruitment

A large-scale, online, cross-sectional research design was used to collect qualitative survey data. The qualitative survey design is an effective method for collecting data that covers potentially sensitive topics, as participants benefit from greater anonymity and privacy, and is also ideal for collecting data from a large, international sample (27). A total of 1,983 women who self-reported experiencing rUTI were recruited for participation between July 2019 and June 2021. Self-reported UTI symptoms are important indicators for UTI diagnosis, alongside or independent of the results of routine (culture-based) testing methods, with self-report of rUTI therefore considered a valid screening tool (28). Participants were between the ages of 18 and 99; 55.3% were from the United States, and 19.3% were from the United Kingdom (see **Table 1**). Participants were included in the analysis if they met the following inclusion criteria: female, over the age of 18 years, and self-reported number and frequency of UTI episodes met the most commonly accepted definition of rUTI (i.e., experiencing ≥ 2 UTIs in the previous 6 months, or ≥ 3 in the previous 12 months) (7, 8). The exclusion criteria were the non-disclosure of UTI and not meeting the classification criteria for rUTI. Participants were not excluded on the basis of disclosure of other medical diagnoses, as it is common for people living with rUTI to be living with comorbid conditions (29); such sampling therefore allowed the rUTI patient population to be represented more accurately.

**Table 1:** Sample characteristics.

Demographics		*N*	%
Age (years)	18–24	426	21.5
	25–29	346	17.4
	30–39	354	17.9
	40–49	262	13.2
	50–59	216	10.9
	60–69	227	11.4
	70–79	129	6.5
	80–89	22	1.1
	90–99	1	0.1
Country	United States	1,116	56.3
	United Kingdom	383	19.3
	Canada	105	5.3
	Australia	90	4.5
	Other	289	14.6
Total number of UTIs experienced in lifetime	2–6	525	26.5
	≥7	1,458	73.5

### 2.2 Materials

A brief online survey was used to collect demographic information (age, sex, and country), UTI-related information (i.e., the number and frequency of UTIs, UTI symptoms, and information related to other medical diagnoses), and information about whether participants experienced an impact on any of the following: sleep, normal work, activities of daily living, ability to exercise and/or maintain a healthy lifestyle, enjoyment of life, enjoyment of favorite activities, mental health, finances, relationships with friends and family, relationships with partners, or sexual behavior. These items were determined to be the quality-of-life factors most likely to be impacted by rUTI, according to existing research (10, 12, 17). Qualitative responses were then gathered via a free-text field in response to the follow-up question “Are there any factors around recurrent UTI we have not considered that you feel are important?”, which sought to expand upon the multiple-choice questionnaire regarding participants’ quality of life. Open survey questions are regarded as a valuable data collection method for women’s health research as they allow respondents to corroborate their responses to preceding questions and provide an opportunity for researchers to detect important issues that could not be identified with a purely quantitative design (30).

### 2.3 Procedure

Visitors to a website hosted by a patient advocacy organization, Live UTI Free, were invited to complete the rUTI survey. Consent was confirmed through electronic acknowledgment of the “Privacy Policy” and “Terms and Conditions”, which were accessible prior to survey completion. Participants could withdraw from the survey at any time by closing their browser. On completion of the survey, participants were provided with personalized rUTI education information that was generated based on their responses. Before participant data were downloaded for use in the study, ethics approval was obtained from the University of Buckingham School of Psychology and Wellbeing Ethics Committee. All data were anonymous and stored confidentially in accordance with GDPR procedures.

### 2.4 Data analysis

Participant comments were imported into NVivo data analysis software (v.12). The data were analyzed using the “coding reliability” approach to thematic analysis (TA) (see **Table 2** for details of the analytic steps) (32). This approach is recommended for the systematic assessment of large data sets, providing a more objective and structured stance that reduces researcher bias, and identifies the most salient patterns within the responses (33). Between 1 and 744 words were provided per participant, with the average response being 15 words (SD = 23.46).

**Table 2:** Thematic analysis (coding reliability) steps.

Step	Description
1. Initial code sources	The first researcher KM familiarized themselves with the data by reading through the survey responses multiple times and noting items of potential interest in a reflective log.
2. Initial code development	The entire data set was coded inductively, with conceptually similar codes grouped into secondary codes. While searching for the themes, central organizing concepts were considered in light of the overall narrative of the data.
3. Codebook development	The representative themes were then grouped under two overarching themes, reviewed, and finalized using a thematic map. The first and last researchers KM and KF discussed the themes in light of the thematic map, and these were defined and named. The first researcher created and refined a codebook, which included the themes, codes, descriptions and examples.^*^
4. Codebook application	The researchers discussed the codebook and then applied it to a small subset of survey comments (*n *=* *28). Discrepancies were discussed and resolved by the entire research team (*n* = 6 researchers), and the codebook was finalized. The codebook was applied to the data set and generated a high inter-rater reliability score (IRR = 0.96) (31).

^*^See **Additional File 1**.

## 3 Results

**Figure 1:**
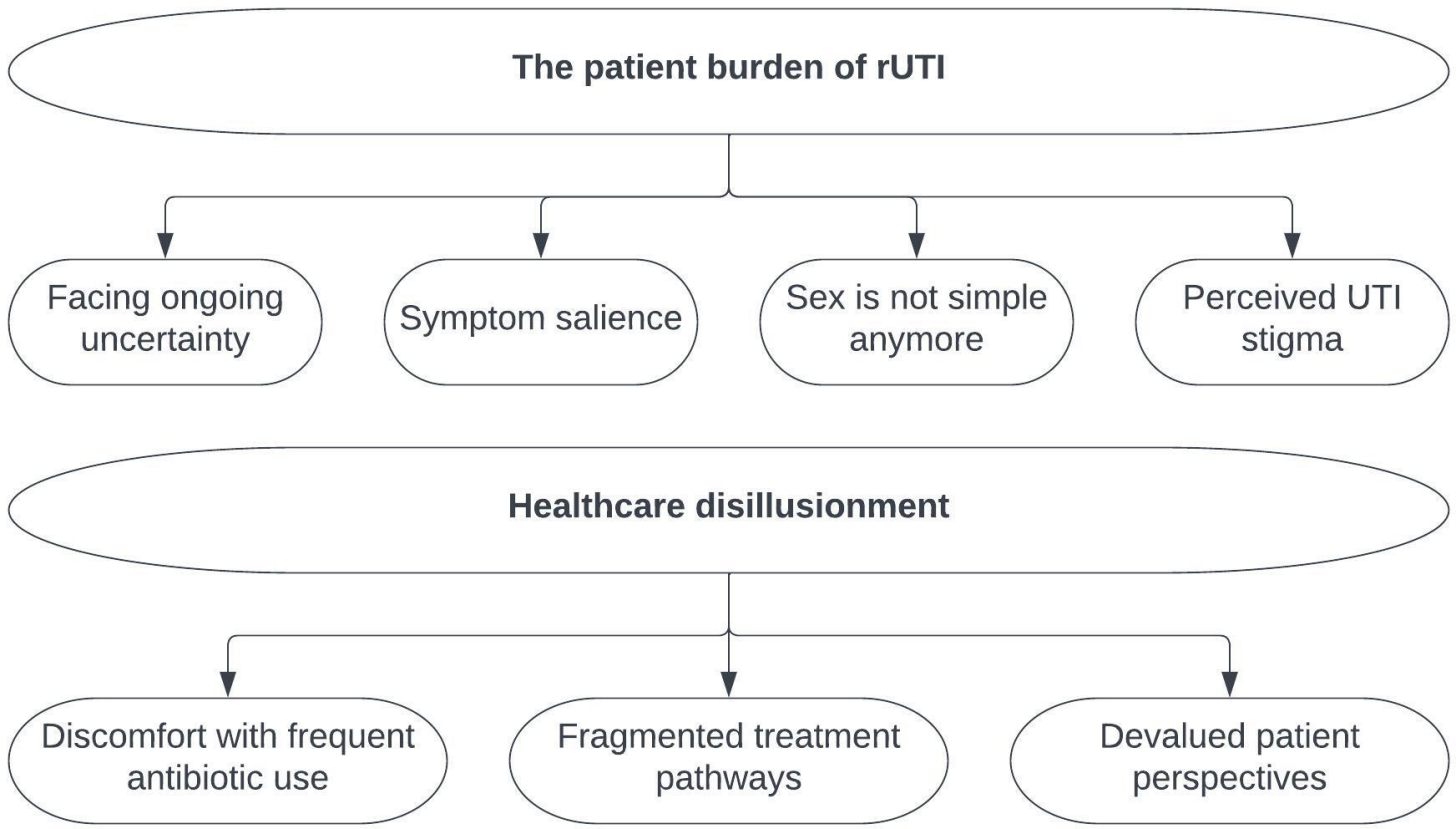
Thematic map.

Two overarching themes were evident within the data: (1) the patient burden of rUTI, which describes the multifaceted biopsychosocial impact of the illness, and (2) healthcare disillusionment, which describes patient dissatisfaction with the healthcare received, both in terms of the treatments offered and communication with healthcare professionals. The thematic analysis identified seven subordinate themes, which fell under the overarching themes (see **Figure 1**): (1.1) ongoing uncertainty; (1.2) symptom salience; (1.3) sex is not simple anymore; (1.4) perceived stigma around UTIs; (2.1) discomfort with frequent antibiotic use; (2.2) fragmented treatment pathways; and (2.3) devalued patient perspectives. A summary of the thematic analysis results can be found in **Table 3**.

**Table 3:** Summaries of each theme with illustrative quotes.

Overarching theme	Subordinate theme	Subordinate theme definition	Example quotes
The patient burden of rUTI	Ongoing uncertainty	Patients placed emphasis on the length of time spent living with rUTI, the uncertainty of when they would next experience symptoms, and the resultant emotional burden.	“[It was] depressing to know I have no control over them occurring any time.” “[The] ongoing cycle of UTI pain.”
	Symptom salience	Patients described the disruptive and overpowering nature of certain recurring UTI symptoms.	“Extreme pain, the worst pain ever. So painful that I cry and scream.” “The onset of my symptoms goes from mild to severe in a matter of a couple hours, which makes getting to the lab for a sample and the pharmacist for an RX [medical prescription] extremely difficult.”
	Sex is not simple anymore	Concerns were raised about trying to conceive amidst recurrent infections, fear of having sex, and subsequently negatively impacted relationships.	“I am planning a baby and I’m always stressed about getting a UTI from intercourse and I feel like that stress is not letting me conceive.” “It’s made me seriously consider giving it up. Which means a lonely, partner-less life.”
	Perceived stigma around UTIs	Hygiene-related stigma, in addition to stigma based on gender and the role of sexual activity in UTI etiology, was felt to be attached to rUTI.	“It makes you feel dirty, like you’ve done something wrong because of the link to sexual intercourse.” “Doctors, in my experience, seem to relate recurrent UTIs to being sexually promiscuous. I have been told before ‘to stop having sex so much’ and to ‘have fewer sexual partners’ with no awareness of my sexual history.”
Healthcare disillusionment	Discomfort with frequent antibiotic use	Antimicrobial resistance, health implications of antibiotic use, and feeling dependent on antibiotics were highlighted by patients as causes for concern with regard to treatment plans.	“The constant treatments with antibiotics since childhood made me immune to a lot of them. On top of that it created problems with bacteria in my gut and an unhealthy immune system. This caused me to constantly become ill and tired. All viruses that came along I became infected with.” “I don’t want to take an antibiotic all the time but I don’t want to let it develop into a worse infection.”
	Fragmented treatment pathways	Issues were identified surrounding inconsistent UTI testing, receiving varying diagnoses, and consequently experiencing inconsistent clinical symptom management.	“I’m soooo tired of being told that my culture came back negative when my symptoms are consistent and very painful. I could scream.” “There really is a need for a better referral system for patients like us, since most doctors don’t know what to do with us and many specialists throw us in the trash bucket of ‘IC’ [interstitial cystitis].”
	Devalued patient perspectives	Patients described problematic interactions with healthcare professionals, and the struggle to have their experience acknowledged and validated.	“One of the most frustrating things is being minimized, disregarded, or laughed off by doctors that I waste my time and money going to in hopes of help. It is SO frustrating to know my own body, know my own symptoms, do so much research, and still be told that I don’t really know what I’m talking about because the test is negative. It is distressing how insensitive some doctors can be to the pain, and how easily they brush off the symptoms as ‘not real’ if the quick pee test doesn’t agree.”

### 3.1 The patient burden of rUTI

Participants disclosed that living with rUTI entailed a significant biopsychosocial burden. Living with this condition was felt to add strain to relationships and negatively affect physical and mental wellbeing.

#### 3.1.1 Facing ongoing uncertainty

Participants placed emphasis on the length of time spent living with rUTI and the resultant emotional burden. The emotional impact of being stuck in an “ongoing cycle of UTI pain” (participant 1,179, United States, aged 25–29 years) was described. Participants stated that it was “depressing to know I have no control over them occurring any time” (participant 869, United States, aged 50–59 years), and that continuing with normal daily life was not possible when living with the uncertainty of when symptoms would next occur.

In addition to more immediate struggles, many participants also felt fearful of and hopeless about their future because of the prospect of never feeling well again. They felt “trapped” (participant 1,259, Canada, aged 70–79 years), “frustrat[ed] at not being able to be ‘fixed’ ” (participant 445, Portugal, aged 50–59 years), and had “the feeling that I’ll never be ‘normal’ again” (participant 1,857, United States, aged 40–49 years). These comments demonstrate that the uncertainty of living with rUTI has serious implications for the psychological wellbeing of patients.

#### 3.1.2 Symptom salience

The salience of physical symptoms was strongly evident as participants discussed the implications of facing repeated cycles of UTI symptoms. These included UTI-related pain and feeling a constant urge to urinate. For instance, one participant reported feeling “extreme pain, the worst pain ever. So painful that I cry and scream” (participant 406, United Kingdom, aged 18–24 years). Urinary urgency in particular was felt to impede daily activities. This meant feeling “afraid to leave my house because I always feel like I need the loo” (participant 623, United Kingdom, aged 25–29 years), or “the constant terror over ‘am I leaking? Did I wait 5 minutes too long to pee?’ ” (participant 1,943, United States, aged 30–39 years).

It was also noted that symptom severity could escalate rapidly, making it more challenging to get help to resolve the UTI: “The onset of my symptoms goes from mild to severe in a matter of a couple hours, which makes getting to the lab for a sample and the pharmacist for an RX [medical prescription] extremely difficult” (participant 1,349, United States, aged 30–39 years). This is demonstrative of the overpowering nature of recurrent UTI symptoms. It is evident that patients’ lives are completely disrupted until symptoms subside, and this can have a devastating effect on their quality of life.

#### 3.1.3 Sex is not simple anymore

The focus in this theme shifts from the individual to the relational and familial impact of rUTI, and we explore how participants dealt with their sex lives being impaired. Concerns were raised about how rUTI impacts trying to conceive, embedding a fear of having sex, and causing relationship tension with partners. UTIs were felt to hinder attempts to conceive: “I am planning a baby and I’m always stressed about getting a UTI from intercourse and I feel like that stress is not letting me conceive” (participant 736, Canada, aged 18–24 years). Participants also described the relational impact stemming from a fear that sex would trigger recurrence: “I know sex causes them and I often don’t want to have sex for the chance I’ll get a UTI so this strains my relationship” (participant 398, United States, aged 40–49 years). The fear was felt by partners as well: “my partner is afraid of having sex with me for fear of my frequent UTIs” (participant 95, United States, aged 25–29 years). It was clear that the link between UTIs and declining sexual wellbeing presented as a significant stressor for women, to the extent that complete abstinence was considered: “it’s made me seriously consider giving it up. Which means a lonely, partner-less life” (participant 1,713, United States, aged 60–69 years). This negative impact of rUTI on starting a family and maintaining intimate relationships was a recurring theme.

#### 3.1.4 Perceived UTI stigma

Healthcare providers and other sources of information were perceived to be stigmatizing UTIs as a result of intertwining the concepts of hygiene and sex with the etiology of UTIs. Participants perceived that they were subject to different types of stigma, which affected them personally as this caused them to “feel like it’s my fault” (participant 335, United States, aged 70–79 years). For the most part, the stigma identified was related to personal hygiene: “It makes you feel dirty, like you’ve done something wrong because of the link to sexual intercourse” (participant 136, United Kingdom, aged 25–29 years); “When researching causes, so many seem to indicate that you’re to blame: clean properly after sex, use the toilet after sex, drink enough, practice good hygiene, etc.” (participant 199, United Kingdom, aged 25–29 years). However, participants additionally identified stigma around promiscuity:

“Doctors, in my experience, seem to relate recurrent UTIs to being sexually promiscuous. I have been told before ‘to stop having sex so much’ and to ‘have fewer sexual partners’ with no awareness of my sexual history. I find doctors assume that and then basically brush it off” (participant 1,133, United Kingdom, aged 18–24 years).

Gender-based stigma exhibited by medical professionals was also identified, with concerns raised about “an undercurrent of deep-rooted sexism certainly play[ing] a role in the pathetic lack of understanding of such a common and potentially serious ailment” (participant 1,624, United States, aged 40–49 years). Therefore, perceived stigma from family, healthcare professionals, and wider society collectively contributed to the psychosocial burden experienced by participants.

### 3.2 Healthcare disillusionment

The second overarching theme was healthcare disillusionment. Participants detailed adverse experiences with their healthcare, which encompassed the challenges faced in receiving a diagnosis, persistently navigating unsuccessful treatment attempts, and communication barriers when conversing with healthcare professionals.

#### 3.2.1 Discomfort with frequent antibiotic use

Participants raised concerns about antimicrobial resistance and the amount of time spent taking antibiotic treatment. A conflict was evident between the need for antibiotic treatment to resolve debilitating rUTI symptoms, and concerns about dependency. Fear about antibiotic-related health risks included concerns about “the effects of long-term antibiotic use, such as constipation, yeast infections, GI [gastrointestinal] issues” (participant 602, United States, aged 30–39 years). Physical health was also thought to suffer as a result of repeat antibiotic prescriptions:

“The constant treatments with antibiotics since childhood made me immune to a lot of them. On top of that it created problems with bacteria in my gut and an unhealthy immune system. This caused me to constantly become ill and tired” (Participant 240, the Netherlands, aged 30–39 years).

The feeling of needing but not wanting antibiotics was strongly highlighted: “I don’t want to take an antibiotic all the time but I don’t want to let it develop into a worse infection” (participant 685, United States, aged 18–24 years) and “I am afraid I will become immune to any antibiotic treatment after that, but the pain is too big when an episode happens” (participant 846, Guatemala, aged 18–24 years). The treatment of rUTI episodes individually without consideration for long-term implications caused participants considerable psychological stress and physical discomfort.

#### 3.2.2 Fragmented treatment pathways

This theme focused on the issues surrounding UTI testing methods, receiving varying diagnoses, and consequently experiencing inconsistent clinical symptom management. Participants indicated that UTI testing methods provided inconsistent results, and some had received misdiagnoses during their illness journey. Negative standard culture test results were received, which conflicted strongly with patient-reported symptom experiences: “I’m soooo tired of being told that my culture came back negative when my symptoms are consistent and very painful. I could scream” (participant 334, United States, aged 50–59 years). This highlighted a disconnect between patient self-report and culture validation.

Due to the confusion around test results, some participants had received diagnoses other than rUTI, for example: “I was misdiagnosed with PBS [painful bladder syndrome] and it transpires I had a UTI for 6 whole years before a doctor finally gave me a 6-month course of antibiotics” (participant 935, United Kingdom, aged 40–49 years).

“There really is a need for a better referral system for patients like us, since most doctors don’t know what to do with us and many specialists throw us in the trash bucket of “IC” [interstitial cystitis].” (participant 391, United States, aged 30–39 years).

Therefore, even before receiving antibiotic treatment, the testing and diagnosis processes for patients exhibiting recurrent UTI symptoms can be fragmented, and cause patients to become disillusioned with the healthcare they receive.

#### 3.2.3 Devalued patient perspectives

Patient–HCP interactions were often characterized as problematic. Participants felt that HCPs were unsympathetic and dismissive, and their lack of trust in the patients’ self-reported experience was described in detail:

“One of the most frustrating things is being minimized, disregarded, or laughed off by doctors that I waste my time and money going to in hopes of help. It is SO frustrating to know my own body, know my own symptoms, do so much research, and still be told that I don’t really know what I’m talking about because the test is negative. It is distressing how insensitive some doctors can be to the pain, and how easily they brush off the symptoms as ‘not real’ if the quick pee test doesn’t agree” (participant 1,677, United States, aged 50–59 years).

Some participants had found doctors who were well informed about UTI recurrence: “I was pleasantly surprised about my doc being pretty clued up. He agrees with the biofilm theory” (participant 1,679, South Africa, aged 50–59 years). For others, the feeling of being dismissed was described in a number of ways, including not feeling “understood or heard, especially when I tell them it’s recurrent” (participant 268, Canada, aged 18–24 years) and “the gaslighting that comes when you test negative for a UTI yet still have symptoms” (participant 1,236, US, aged 50–59 years). These reports demonstrate the extent to which rUTI patients feel that their perspectives are undervalued by clinicians, an experience which left these participants feeling frustrated and distressed. Thus, declines in psychological wellbeing were exacerbated by negative interactions with HCPs.

## 4 Discussion

In summary, the thematic analysis of the data revealed two overarching themes: (1) the patient burden of rUTI, which consisted of the subordinate themes, facing ongoing uncertainty, symptom salience, sex is not simple anymore, and perceived UTI stigma; and (2) healthcare disillusionment, including discomfort with frequent antibiotic use, fragmented treatment pathways, and devalued patient perspectives. These themes elucidate the illness-related factors which are most salient to people living with rUTI and which affect their day-to-day life.

The patient burden associated with rUTI was clearly evident in the results. UTI symptoms have been established as severe and bothersome and have been associated with negatively impacted mental health, sex life, social function, and daily activities (11, 12, 14, 34, 35), as evidenced by the findings of this study. Furthermore, perceived hygiene-related stigma was strongly evidenced, supporting the findings of prior research on such stigma in bladder and bowel patients (36). Evidence of further stigmatization relating to promiscuity compounds the shaming of people living with rUTI. This stigmatization is presented as a predominantly female-specific burden, built through an emphasis on the role of frequent sexual activity in female UTI (37, 38). Lastly, the novel finding regarding the ongoing uncertainty faced by rUTI patients is important as it highlights that it is not only the physical manifestation of the illness but also the impact of uncertain medical prognosis which can be disruptive. This supports evidence that illness uncertainty can cause psychological distress in patients (39). Due to the lack of effective long-term treatment options for rUTI and unpredictable recurrence rates, illness uncertainty may contribute to the deterioration of mental health, adding to the physical burden of the condition. Health psychology research suggests that equipping patients with psychoeducation and emotion regulation techniques could improve the perceived manageability of illness uncertainty (40, 41). Distress was exhibited by patients in this study; therefore, it is clear that the ongoing uncertainty associated with rUTI requires urgent attention from a multidisciplinary clinical perspective.

The second prominent overarching theme was healthcare disillusionment, which is as yet unexplored in the literature. The possible implications of frequent antibiotic use were of major concern, highlighting that rUTI patients want to be able to have discussions with their HCPs about alternative long-term treatment plans (14, 15). The implications of inconsistent testing and diagnosis were also evident, as rUTI patients reported confusing diagnostic approaches; for example, receiving an initial diagnosis of IC/PBS followed by a revised diagnosis of rUTI years later. Devalued patient perspectives carried substantial weight in the present findings, as healthcare professionals were perceived to be unsympathetic and to prioritize professional opinion over patient report. When doctors exhibited a greater understanding of the issue, patient experiences were more positive, and the testing and treatment choices made in these cases may be more conducive to better health outcomes. However, participants in this study described how patient–HCP interactions can feel accusatory, particularly for women, adding to patient burden. HCP communication style can make a significant difference in patient outcomes (42). Recent evidence shows that HCPs understand the importance of managing patient expectations, concerns, and emotional responses when faced with complex decision-making around the medical diagnosis and treatment of rUTI (43). Unfortunately, however, women in particular report low satisfaction with the interactions they have with HCPs, which is indicative of gender-based stigma (18, 24). Although fragmentation exists around the long-term management of rUTI and HCPs may struggle for solutions, communication styles that are more person-centered, consider patient preferences, and are less dismissive or accusatory are more likely to result in better illness management (42). Such communication would also better reflect guidance to adhere to the principles of patient-centered care by valuing individual needs and preferences (44). This would have positive implications for patient wellbeing and potentially encourage more conservative antibiotic use, in turn minimizing the risk factors for antimicrobial resistance.

### 4.1 Implications for practice and/or policy

The findings of this study confirm that rUTI patients experience burden in relation to the following factors: emotional wellbeing, physical functioning, sexual relationships, and social stigma. Burden is exacerbated by diagnostic inconsistency, poor HCP–patient communication, the side effects of frequent antibiotic use, and restrictive testing. To lessen the impact of these stressors, effective HCP–patient communication and shared decision-making are crucial, as are focusing on illness management strategies and treating the patient experience holistically. It is also critical for HCPs to initiate discussions around caution with antibiotic treatment, evaluation of recurrence risk factors, and consideration of alternative treatment, as recommended in the current guidelines provided by bodies including the American Urological Association and the European Association of Urology (45). Effective communication and standardized exploration of the patient perspective can be aided by using validated rUTI-specific patient-reported outcome measures (PROMs), such as theRecurrent UTI Symptom Scale (RUTISS) and Recurrent UTI Impact Questionnaire (RUTIIQ) (46, 47).

Ultimately, the findings from this large-scale international study present the shared qualitative experiences of people living with rUTI across a multitude of different countries, demonstrating widespread patient burden, and transnational deficits in healthcare approaches provided. Although there is still important research investigating the biological processes of rUTI in terms of the composition and fluctuations of the urobiome, in addition to the efficacy of novel biomedical treatment approaches such as vaccines, bacteriophages, and new antibiotics (48) to be conducted, there is also an urgent need to look beyond biological means of diagnosis and treatment. Patient self-report must be respected by healthcare providers so that patient burden is addressed and treatment pathways are tailored to the needs of the individual.

### 4.2 Limitations and future directions

A key strength of the study was that we were able to obtain perspectives from a large and demographically diverse sample. However, a more in-depth qualitative analysis of this subject could be achieved through the collection of interview data, providing an opportunity for probing with open questions to extend knowledge of the lived experience of patients with rUTI. Establishing a better understanding of the barriers and facilitators to illness burden and effective illness management for people living with rUTI could precede intervention design and implementation, which could target behaviors such as shared HCP–patient decision-making. Future research should also consider how experiencing a larger number of UTIs in their lifetime and a higher frequency of UTIs may impact participants’ reporting of the rUTI experience, particularly in relation to the areas of burden identified. Notably, the findings also reflect the experiences of a self-selecting sample of participants; however, as there is a paucity of research on the rUTI patient experience, and we were able to obtain perspectives from a large, international sample, the results of this study have significant implications for clinical practice.

### 4.3 Conclusions

Living with rUTI is associated with both psychological and physical disruptions to daily life. Patients not only face recurring symptom burden, but they are also challenged by the uncertainty of unresolved illness, in addition to the social implications of an impaired sex life and illness-related stigma. Fragmentation is perceived in healthcare approaches, with testing being reported as unreliable, antibiotic treatment causing unwanted side effects, and patients feeling that their perspectives are often deemed inconsequential. To overcome these barriers, which hinder effective rUTI care, increasing shared decision-making and a multidisciplinary approach to the management of the condition are crucial, so that patients’ psychological distress is reduced and the management of their illness is improved. Overall, the findings from the present study highlight the significant need for a better understanding of the lived experiences of people living with rUTI, and also the demand for more patient-centered care.

## Data availability statement

The raw data supporting the conclusions of this article will be made available by the authors, without undue reservation.

## Ethics statement

The studies involving humans were approved by The University of Buckingham School of Psychology and Wellbeing Ethics Committee. The studies were conducted in accordance with the local legislation and institutional requirements. The participants provided their written informed consent to participate in this study.

## Author contributions

KM: Conceptualization, Formal analysis, Methodology, Project administration, Writing – original draft, Writing – review & editing. LR: Conceptualization, Formal analysis, Methodology, Supervision, Writing – review & editing. MK: Conceptualization, Data curation, Formal analysis, Resources, Supervision, Writing – review & editing. JP: Conceptualization, Formal analysis, Writing – review & editing. AN: Conceptualization, Formal analysis, Writing – review & editing. KF: Conceptualization, Formal analysis, Methodology, Supervision, Writing – review & editing.
