# Supplementary material for: Psychosocial burden and healthcare disillusionment in recurrent UTI: a large-scale international survey of patient perspectives
Source: Front Urol. 2023 Sep 20;3:1264299. doi: 10.3389/fruro.2023.1264299 (PMC12327290; doi:10.3389/fruro.2023.1264299)
Supplement: Supplementary file 1 [file DataSheet_1.pdf]

Overarching theme	Theme	Theme definition	Inclusion / exclusion criteria	Codes	Examples
The patient burden of rUTI	Facing ongoing uncertainty	This involves participants feeling hopeless about their present situation, scared about future health complications, and desperate for a return to normality	Includes any mention of depressive symptoms / losing hope / desperation / fearing neverending health problems	Exhibiting depressive symptoms	"I really think that this has made me at times suicidal"
				Making future plans is impossible	"can really disrupt your life to the extent that you don't want to plan anyhting "in case" you get a UTI"
				Fear of recurrence	"Constantly worrying about getting the next one. And everytime I go to pee I'm making sure if I feel any of the symptoms, even just a little tingling and I start to panic"
				Wanting to be healthy again	"Just my health in general. I want to be back to normal"
	Symptom salience	Symptoms make it hard to continue with normal daily activities; UTI symptoms are extremely unpleasant and symptoms of a yeast infection or treatment side effects add to the symptom burden; Focus is put on correlating symptoms with what is happening internally	Includes daily activities which are disrupted due to symptoms, comments on symptom severity / bothersomeness, symptoms from comorbid issues, pinpointing bacterial species behind the infection causing the symptoms	Depending on toilet facilities	"The difficulties of going out, not knowing where to find toilets"
				Impacted sleep and fatigue	"Passing urine many times at night causing lack of sleep effecting daytime activities"
				Life put on hold	"I avoid early morning commitments and have had to cancel later appts (including walking out of one) at times"
				The pain is debilitating	"I just get crazy with not being able to get rid of the pain."
				Physical discomfort	"I can't find a comfortable position, I am either sitting on the toilet or pacing"
				UTI - yeast infection symptom cycle	"My UTIs are always followed by yeast infections when I start treatment. It's a viscous cycle"

Overarching theme	Theme	Theme definition	Inclusion / exclusion criteria	Codes	Examples
				Antibiotic side effects	"When I'm prescribed antibiotics for these UTI's the side effects from them affect my daily life which as another factor. Sometime I live with a UTI and don't bother getting antibiotics because the side effects from the medication are so bad aswell"
				Searching for symptom patterns	"Symptoms seem to differ (from bladder pain to bladder pressure to pain urinating to blood in urine) every month"
				Escalating symptom severity	"I feel like it's holding me back, I will be compltely fine and then within 10 minutes im suddenly ill, and can't do much for the next 2 days"
	Sex is not simple anymore	Sex life is impacted by fear of infection, TTC stress; Negatively affected sex life impacts relationships	Includes mention of sexual relationships negatively affected, TTC worries, fear that sex will trigger an infection	Implications for sex life	"I get UTI almost evry time I have sex, to the point where I choose not to have sex with my partner because I don't want to go through the pain"
				Fear of having sex	"I want SOLUTIONS to never having one again. So I can be intimate with my husband and express my love and appreciation for him, without living in fear that I'm going to get yet again another UTI from sex"
				Impacts TTC	"How to conceive when you get reoccurring UTIs. What to do if you're trying to conceive and get one"
	Perceived UTI stigma	Participants perceived judgement from others about the cause of UTIs, especially with regards to hygiene and promiscuity, and felt misunderstood by others	Includes mention of stigma, feeling unheard / misunderstood / excluded by others (friends / family / doctors) because of misconceptions, internalising stigma	Others don't understand	"It is an awkward thing to talk about so hard for people to understand"

Overarching theme	Theme	Theme definition	Inclusion / exclusion criteria	Codes	Examples
				Stigma of poor personal hygiene	"My mom seems to think that the only reason I get UTIs often is because I don’t clean myself properly after sex (which is not true). I’ve stopped mentioning them to her because she always makes comments like that"
				Stigma of promiscuity	"Doctors, in my experience, seem to relate recurrent UTIs to being sexually promiscuous. I have been told before "to stop having sex so much" and to "have less sexual partners" with no awareness of my sexual history"
				Self-blame	"I feel guilty that I can't seem to find a way to stop them, and somewhat stupid"
				Impacted self-image	"I feel extremely unattractive when I feel my daily reoccurring BV and UTIs I get every few months"
				Perceived health inequality	"Doctors keep telling me it is normal to have a UTI ever 3-6 months because I am a woman"
Healthcare disillusionment	Discomfort with frequent antibiotic use	This involves participants questionning the use of antibiotics in terms of the health implications and fear of becoming resistant. Participants expressed discomfort with dependence given these two concerns, and described their attempts with alternative treatment / prevention options	Includes comments about antibiotics causing other health issues, mention of AMR, descriptions of alternative strategies tried	Needing but not wanting antibiotics	"I don’t want to take an antibiotic all the time but I don’t want to let it develop into a worse infection"
				Antibiotic health risk	"On top of that it created problems with bacteria in my gut and an unhealthy immune system. This caused me to constantly become ill and tired. All viruses that came along I became infected with. Couldn’t function normally anymore"

Overarching theme	Theme	Theme definition	Inclusion / exclusion criteria	Codes	Examples
				Choosing non-antibiotic treatment	"I don't want to take antibiotics. Also Chinese medicine and acupuncture helps alot"
	Fragmented treatment pathway	The treatment pathway from testing and diagnosis to treatments prescribed or recommended is ineffective, as participants do not get the care that they need, UTIs always return, and the problem is never resolved	Includes any mention of not having good access to healthcare, testing failure, treatment failure, lacking a long-term plan	Test results are inconsistent / inaccurate	"How sometimes the test says you don't have one and the very next day you go somewhere else to get tested and they tell you you do have one"
				Need for knowledge	"I feel like there's not enough research on chronic UTIs. Every time I go in to the doctors they ask me the same questions: do you wipe front to back, do you pee after sex, do you shower every day? No one in the medical profession can make me feel better when it comes to why I have been getting them since I was 12"
				Wanting a more holistic treatment plan	"Maybe a plan of action for what choices/options and risks in a clear step by step summaryfor how to fight A uti. I'm still unsure what risks I have when I use antibiotics over and over again and if I don't take them then how long should I wait to try treating it naturally before I should go see a doctor"
				Treatment failures	"When I have a uti I get another one 5 days after ending course if antibiotics. This for 5 years"
				IC vs. UTI confusion	"Never sure if it's an I.C. flare up or infection. Not even sure if I was d iagnosed properly in reference to the I.C"
				Possible embedded infection	"It's a constant struggle to keep it away because something is living inside my bladder!"

Overarching theme	Theme	Theme definition	Inclusion / exclusion criteria	Codes	Examples
				Seeking treatment is time-consuming	"My life is totally disrupted, I have had 10-12 infections in the last 5 1/2 months. That is real and it amounts to an infection almost every other week."
	Devalued patient perspectives	Participants experience relationship strain with doctors as they percieve that doctors not not care / are unwilling to help / do not trust their reported experience / would rather ignore them	Includes any comment on a negative experience with a HCP	Distrust in patient report	"the frustration of doctors who don't listen or believe something is wrong when you describe to them your symptoms"
				Doctors are dismissive	"It has been frustrating to be treated seriously when I try and go to the doctor. Most ignore my complaints. Some won't even test and tell me to just drink more water"
				Wanting an empathetic doctor	"I need to see a good practitioner that understands my issues and will advocate for me"
